# Phytochemical Analysis and Appraisal of Antiproliferative Activity of *Magnolia alejandrae*

**DOI:** 10.3390/metabo15090567

**Published:** 2025-08-22

**Authors:** José E. Caballero-Chávez, Alma D. Paz-González, Diana V. Navarrete-Carriola, Fabián E. Olazarán-Santibañez, María Miriam Estevez-Carmona, Benjamín Nogueda-Torres, Fernando Emiliano Jiménez-Mondragón, Melany X. Márquez-Aguilar, Carmen Michelle Pineda-Alcala, Diego Cisneros-Juárez, Álvaro Marín-Hernández, Debasish Bandyopadhyay, Gildardo Rivera

**Affiliations:** 1 Laboratorio de Biotecnología Farmacéutica, Centro de Biotecnología Genómica, Instituto Politécnico Nacional, Reynosa 88710, Mexico; jcaballeroc2200@alumno.ipn.mx (J.E.C.-C.); apazg@ipn.mx (A.D.P.-G.); dnavarretec1900@alumno.ipn.mx (D.V.N.-C.); 2 Facultad de Medicina Veterinaria y Zootecnia, Universidad Autónoma de Tamaulipas, Ciudad Victoria 87000, Mexico; feolazaran@docentes.uat.edu.mx; 3 Escuela Nacional de Ciencias Biológicas, Instituto Politécnico Nacional, Ciudad de México 07320, Mexico; mmestevez@ipn.mx (M.M.E.-C.); bnogueda@ipn.mx (B.N.-T.); 4 Departamento de Bioquímica, Instituto Nacional de Cardiología Ignacio Chávez, Ciudad de México 14080, Mexico; emi1920@comunidad.unam.mx (F.E.J.-M.); 317070589@iztacala.unam.mx (M.X.M.-A.); carmenmichelle.pineda@ciencias.unam.mx (C.M.P.-A.); dieguito@ciencias.unam.mx (D.C.-J.); alvaro.marin@cardiologia.org.mx (Á.M.-H.); 5 School of Integrative Biological and Chemical Sciences (SIBCS), University of Texas Rio Grande Valley, Edinburg, TX 78539, USA; debasish.bandyopadhyay@utrgv.edu; 6 School of Earth, Environmental, and Marine Sciences (SEEMS), University of Texas Rio Grande Valley, Edinburg, TX 78539, USA

**Keywords:** antiproliferative activity, cancer cell lines, chromatography, *Magnolia alejandrae*, secondary metabolite profile

## Abstract

**Background:** *Magnolia alejandrae* is a tree endemic to Tamaulipas, Mexico, distributed in the forests of the Sierra Madre Oriental. **Objective:** Our objective was to analyze the secondary metabolite profile of different parts of *M. alejandrae* and evaluate their antiproliferative activity in vitro. **Methods:** Different extracts of leaf, bark, and fruit were obtained using conventional and unconventional extraction methods with solvents of different polarity. The extracts were analyzed by Ultra-Performance Liquid Chromatography-Mass Spectra (UPLC-MS), and their antiproliferative activity against cancer cell lines was determined. **Results:** The primary yields of the extracts obtained from *M. alejandrae* ranged from 8.32% to 36.19%. Three hundred and twelve secondary metabolites previously reported from the Magnolia genus were detected. The most frequent were magnone A, pinoresinol, and yangambin. Honokiol and magnolol were not detected. Two of the extracts (FSW and BSW) had antiproliferative activity (IC_50_ < 140 µg/mL) against HeLa, MCF-7, A549, U373, and PC3 cancer cell lines. The higher activity was against the A549 cell line. **Conclusions:** *M. alejandre* extracts showed secondary metabolites previously reported and unreported in other species. Interestingly, some extracts had antiproliferative activity against cancer cell lines. Therefore, *M. alejandrae* is a source of molecules that could be explored to develop new drugs.

## 1. Introduction

The Magnolia genus has a longstanding role in traditional Asian medicine. Different Magnolia species, such as *M. officinalis*, *M. stellata*, *M. grandiflora*, and *M. coco,* have been widely used for various disorders such as headache, fever, anxiety, diarrhea, stroke, and asthma, sleep disorders, and allergic diseases [1,2,3]. Additionally, Magnolia extracts have been reported with different biological properties such as antimicrobial, anti-inflammatory, antioxidant, anticancer, antianxiety, and antiproliferative. These therapeutic properties have been attributed to the presence of numerous secondary metabolites, including terpenes, polyphenols, and alkaloids [4,5,6,7,8,9,10].

Honokiol, magnolol, 4-O-methylhonokiol, and obovatol are considered major bioactive constituents of Magnolia stem bark, belonging to the phenylpropanoid group, specifically neolignanes, which are notable for their antimicrobial and anticancer properties. Different studies report that the antiproliferative activity of Magnolia extracts is also mainly due to the presence of other secondary metabolites such as alkaloids, flavonoids, terpenoids, and polyphenols [10,11,12,13]. Due to the presence of these compounds in Magnolia extracts, various studies have been conducted to evaluate their antiproliferative activity against different cancer cell lines, for example, the methanolic extract of *M. officinalis* bark at a concentration of 20 to 50 µg/mL induces apoptosis in HeLa cells by activating the intrinsic apoptosis pathway, in addition to inhibiting proliferation in A549 and PC3 cells [14]. Similarly, the ethanolic extract of *M. denudata* flowers at 125 µg/mL caused a 68.7% inhibition against the MCF-7 breast cancer cell line [15,16,17].

In Mexico, different species of Magnolia have been reported in recent years (2017)*. M. alejandrae* was discovered with an endemic distribution in the northeastern region in a restricted area of occupancy and a limited presence; its population density is estimated at 500 individuals per hectare (ha) with a total area of 23 ha [18,19]. *M. alejandrae* lives exclusively within the cloud forests and humid pine and oak forests of the Sierra Madre Oriental in Tamaulipas, Mexico, which are subject to anthropogenic disturbances and climate change, as well as agricultural activity and the effects of drought years [20]. These factors could affect their chemical composition and biological properties. Recently, our research group explored the qualitative chemical composition and acaricide activity of leaf ethanolic extracts of *M. alejandrae* [21]. However, there is a need for studies more specific about the phytochemical composition of each part of the plant, the appropriate extraction method, and the solvent, considering the kind of secondary metabolites and expanding their potential biological properties.

The objective in this study was to determine the secondary metabolite profile in the fruit, leaves, and bark of *M. alejandrae* using various solvents and extraction methods. Additionally, we aimed to explore the antiproliferative activity of the extract obtained and the secondary metabolites, magnolol and honokiol, against various cancer cell lines.

## 2. Materials and Methods

### 2.1. Collection and Identification of Plant Material

*M. alejandrae* was collected in Los San Pedros, Güémez, Tamaulipas, Mexico (123°51′50.457″ N, 99°23′18.751″ W), in June 2023. The plant material was collected from 20 adult specimens from the same population. Fruits, leaves, and stems were disinfected with a commercial detergent solution (1% *w*/*v*) and dried at 60 °C for 3 days; the bark was removed from the stems. The plant material was pulverized and stored in the dark at room temperature until its use. The collected specimens meet the diagnostic traits of *M. alejandrae* described by García Morales et al. in 2017 [22]. The identification of the specimens was confirmed by direct comparison with the original description and topotype 22754. This identification was made by Dr. Manuel Yáñez Pacheco (Francisco González Medrano Herbarium from the Instituto de Ecología Aplicada, Universidad Autonoma de Tamaulipas, Mexico), an expert in Magnoliaceae taxonomy.

### 2.2. Extraction Procedure

The secondary metabolites were extracted from pulverized plant parts using four methods (maceration, Soxhlet, reflux, and ultrasound) and three solvents dichloromethane, ethanol, and water (Sigma Aldrich, Toluca, Mexico) in triplicate. Two grams of plant material were used in all extraction methods. Maceration: 50 mL of solvent was added to the plant material and stored for 8 days at room temperature in the dark. Soxhlet: 100 mL of solvent was added and placed in the Soxhlet apparatus for 6 h. Reflux: 50 mL of solvent was added to the plant material and subjected to reflux for 5 h. Ultrasound: 12 mL of solvent was used and subjected to a Brasonic^®^ ultrasonic bath (Danbury, USA) for 40 min. In the maceration, Soxhlet, and reflux procedures, a filtration process was carried out, and the crude extract was recovered. The organic solvent (dichloromethane and ethanol) was removed under reduced pressure using a BUCHI Rotavapor R-100 (BuchiLabortechnik AG, P.R. of China). Water was removed for 24–48 h in a Labconco TM 77540-00 freeze dryer (Labconco Corporation, Kansas City, USA). The extracts were stored in the dark at 4 °C until use.

### 2.3. Analysis by Ultra-Performance Liquid Chromatography (UPLC-MS)

The Sigma-Aldrich (Toluca, Mexico) standards (honokiol and magnolol) at concentrations of 0.0125, 0.025, 0.05, 0.1, 0.2, 0.4, 0.6, 0.8, and 1 mg/mL in ethanol were vortexed for 1 min and then filtered through a 0.45 μm membrane and transferred to standard vials for analysis. Instrumentation and methods: UPLC-MS analysis was performed using liquid chromatography equipment with an ACQUITY UPLC system coupled to a Waters QDa mass detector (Milford, MA, USA) in positive ion mode. An ACQUITY UPLC CORTECS C18 column was used as a 1.6 μm, 3.0 mm × 100 mm column (Milford, MA, USA). Column and autosampler temperatures of 40 and 15 °C were used, respectively. Elution was achieved with 0.1% formic acid (Sigma Aldrich, Toluca, Mexico) in water (Phase A) and acetonitrile (Sigma Aldrich, Toluca, Mexico) (Phase B). The composition of solvents over time was as follows: initial A: 10%, B: 90%; at 5 min, reduced A: 25%, B: 75%; changing at 10 min, A: 10%, B: 90% with a flow rate of 0.3 mL/min and a run time of 15 min. Sample preparation: 5 μL of each extract (1 mg of crude extract/mL) was injected.

The identity assignment of the detected secondary metabolites in *M. alejandre* extracts was performed by comparing the mass fragmentation patterns and molecular weights with *Magnolia* spp. reported in PubChem. Honokiol and magnolol were identified in the extracts by comparing mass spectra and retention times with corresponding standards, and their quantification was based on the results of the calibration curve standards.

### 2.4. Cell Lines and Culture Conditions

Cancer cell lines HeLa, U373, A549, and PC3 were cultured in Dulbecco’s Modified Eagle Medium (DMEM) high glucose (Gibco, Grand Island, NY, USA) supplemented with 10% fetal bovine serum (FBS) (Biowest, Riverside, MO, USA), 100, 000 U/L penicillin, 100 mg/L streptomycin, 250 µg/L amphotericin (Sigma, St Louis, MO, USA), 110 μg/L sodium pyruvate (Sigma, St. Louis, MO, USA), 3.7 g/L sodium bicarbonate (Sigma, St. Louis, MO, USA), and 6 g/L HEPES (Sigma St. Louis, MO, USA), pH = 7.2. MCF-7 was cultured in a Minimum Essential Medium (MEM) (Sigma, St Louis, MO, USA) supplemented with 10% FBS, 100,000 U/L penicillin, 100 mg/L streptomycin (Sigma), 10 µg/mL insulin (Sigma), and 3.7 g/L sodium bicarbonate, pH = 7.2. MCF-10A was cultured in DMEM/F-12 medium (Gibco, Grand Island, NY, USA) supplemented with 5% horse serum (Biowest, Riverside, MO, USA), 0.5 µg/mL hydrocortisone (Sigma, St. Louis, MO, USA), 20 ng/mL EGF (Sigma, St. Louis, MO, USA), 100 ng/mL cholera toxin (Sigma, St. Louis, MO, USA), 10 µg/mL insulin, 100,000 U/L penicillin, and 100 mg/L streptomycin, pH = 7.2. The cells were incubated at 37 °C in a humidified atmosphere with 5% CO_2_ and 95% air. The MCF-7 cell line was initially acquired from the University of Colorado Cancer Center Tissue Culture Core and was obtained from Dr. Paola Maycotte. The rest of the cell lines were obtained from ATCC (American Type Culture Collection, Manassas, VA, USA). Human cell lines were genotyped by the Instituto Nacional de Medicina Genómica (INME-GEN, Mexico City, Mexico), and the analysis showed that the cell lines used shared 87–100% alleles reported by the ATCC original clones for their authentication.

Each cell line was seeded (5 × 10^5^–1 × 10^6^ cells) in triplicate in a 150 mm Petri dish with 20 mL of supplemented medium; after reaching 80–90% confluence, the cells were detached with 0.25% trypsin/1 mM EDTA. The suspension was centrifuged at 300 g for 3 min. The cellular pellet was resuspended in 1 mL of medium. Afterward, cells were counted using a Neubauer chamber with 0.1% trypan blue staining.

### 2.5. In Vitro Evaluation

In 96-well plates, cells (2 × 10^4^ cells/well) were seeded in 100 µL of culture medium in triplicate and incubated for 24 h at 37 °C in a humidified atmosphere with 5% CO_2_ and 95% air to ensure that they adhered to the bottom of the well. After this time, the culture medium was changed. The fresh medium contained doxorubicin (Dx), magnolol, and honokiol (as a positive control) at concentrations of 0, 5, 10, 25, 50, 75, and 150 µM or 0 and 150 and 300 µg/mL of the extracts Leaf–Soxhlet–Water (LSW), Leaf–Soxhlet–Ethanol (LSE), Leaf–Soxhlet–Dichloromethane (LSD), Fruit–Soxhlet–Water (FSW), Fruit–Soxhlet–Ethanol (FSE), Fruit–Soxhlet–Dichloromethane (FSD), Bark–Soxhlet–Water (BSW), Bark–Soxhlet–Ethanol (BSE), Bark–Soxhlet–Dichloromethane (BSD), Leaf–Ultrasonication–Water (LUW), Leaf–Ultrasonication–Ethanol (LUE), and Leaf–Ultrasonication–Dichloromethane (LUD). The plates were incubated for 24 h. At the end of this time, the medium was removed, and the wells were washed three times with 100 µL of non-sterile PBS. Subsequently, 100 µL of cold ethanol was added, and the plates were incubated at −20 °C for 20 min. After this, the ethanol was removed, and the plates were dried for 24 h. Cells that were fixed in the wells were stained with 0.1% crystal violet. To determine the dye content, absorbance was read at 595 nm.

The half-maximal inhibitory concentration (IC_50_) determination involved fitting the dose–response curve data to a second-order exponential decay equation utilizing OriginPro 2024 (V 10.1). Extracts that demonstrated a superior IC_50_ were subsequently re-evaluated with a broader range of concentrations (0, 25, 50, 100, 150, and 200 μg/mL) to enhance the precision of the IC_50_ calculation.

## 3. Results

### 3.1. Extraction Efficiency

**Figure 1:**
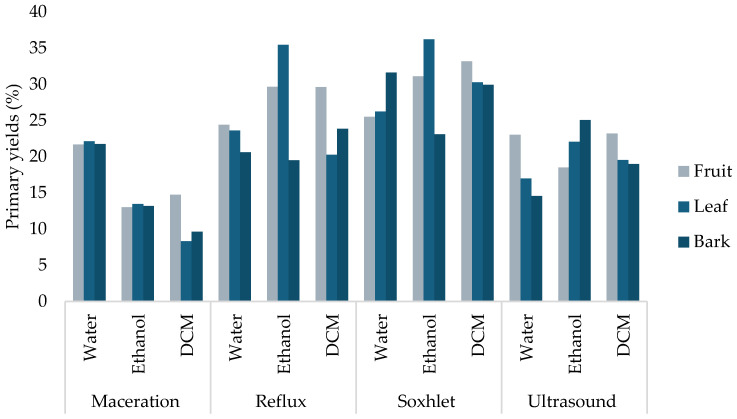
Primary yields (%) of the extracts obtained from *M. alejandrae* using four methods and three solvents. DCM: dichloromethane.

Initially, thirty-six extracts of fruit, leaf, and bark from *M. alejandre* using four methods (maceration, reflux, Soxhlet, and ultrasound) and three solvents (water, ethanol, and dichloromethane) were obtained. The results of the yield are shown in Figure 1, which ranged from 8.32% to 36.19%.

### 3.2. Chromatographic Parameters of Magnolol and Honokiol by UPLC-MS

Initially, the honokiol and magnolol standards were analyzed individually and in proportion (1:1) by UPLC-MS to identify their molecular weight, fragmentation pattern, and retention time (Table 1) under the experimental conditions. Additionally, the calibration curve standards (Supplementary Material, Graphics S1 and S2) were obtained for a potential quantification of both secondary metabolites in the extracts. Finally, the limit of quantification (LOQ) and limit of detection (LOD) were determined (Table 1).

### 3.3. Comparative Analysis of Secondary Metabolites from M. alejandre

**Figure 2:**
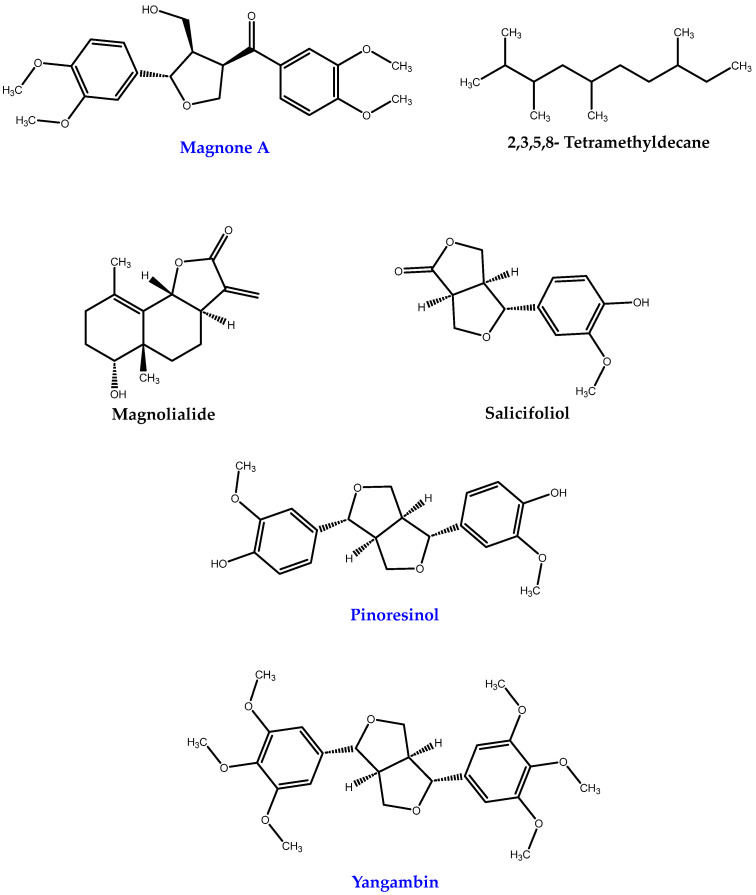
Structures of the most frequent secondary metabolites detected in *M. alejandrae* extracts by UPLC-MS.

The analysis of the secondary metabolites in the thirty-six extracts from *M. alejandre* allows the detection of three hundred and twelve molecules (Supplementary Material, Table S1) by UPLC-MS, previously reported from the Magnolia genus in the PubChem database. The most frequent secondary metabolites identified in ≥10 extracts are shown in Table 2. Magnone A, pinoresinol, and yangambin (Figure 2) were the most frequently detected secondary metabolites in 31–33 extracts (Table 2). Honokiol and magnolol were not detected.

Additionally, in the thirty-six extracts from *M. alejandre*, forty-eight secondary metabolites (Supplementary Material, Table S2) were detected that did not correspond to the molecular weights of those already reported from *Magnolia* spp. The secondary metabolites most frequently detected were ten, with a mean intensity ranging from 1 to 3 million µV/s in at least one extract (Table 3).

### 3.4. Antiproliferative Evaluation of M. alejandrae Extracts Against Cancer Cell Lines

In the present study, the antiproliferative activity of nine extracts with high yields (Soxhlet method) from *M. alejandrae* was evaluated against five cancer cell lines, HeLa (cervical), A549 (lung), U373 (glioma), PC3 (prostate), and MCF-7 (breast), and one non-cancer cell line MCF-10A (human mammary gland cells, CRL-10317) (Table 4). Additionally, ultrasound extracts from leaves were evaluated. The compounds magnolol, honokiol, and doxorubicin were used as positive controls. Two of the extracts (FSW and BSW) obtained by the Soxhlet method had antiproliferative activity with an IC_50_ value < 140 µg/mL against all cancer lines (Table 4). The other extracts had no activity at 300 µg/mL (Table 4). Additionally, the LUE and LUD extracts of leaves from *M. alejandrae* obtained using the ultrasound method exhibited antiproliferative activity. Honokiol, magnolol, and doxorubicin had similar IC_50_ values against all cancer cell lines. All extracts and the positive controls had no antiproliferative activity against the MCF-10A cell line, except for doxorubicin.

Finally, the selectivity index (SI) of the four extracts with antiproliferative activity and the positive controls was determined. The results are shown in Table 5.

## 4. Discussion

The isolation and identification of the secondary metabolites with biological activities is very important in the development of new drugs. However, the concentration of these secondary metabolites may vary depending on the part of the plant (bark, leaves, flowers, or roots), matrix, solvent, temperature, pressure, and time used. Additionally, the methods of extraction used, such as maceration, Soxhlet extraction, and ultrasound- or microwave-assisted, may influence the concentration of these secondary metabolites [22]. For example, the concentration of the extracted secondary metabolites decreases with a prolonged time of maceration, possibly due to the precipitation or degradation [23]. Therefore, selecting the appropriate solvent and the optimal technique is crucial for maximizing the yield of secondary metabolites and minimizing their thermal or chemical degradation. Concerning the above, several studies have focused on the selectivity of the extraction method and the use of different solvents to extract specific secondary metabolites [16,24,25].

In this study, extracts of *M. alejandrae* were obtained using three conventional extraction methods (maceration, reflux, and Soxhlet) and one non-conventional method (ultrasound-assisted extraction) to identify the most suitable method for extracting secondary metabolites with potential anticancer activity [26]. The results showed that Soxhlet was the most efficient method to obtain a high amount of extract, followed by reflux, ultrasound, and maceration. The Soxhlet method is part of the classification of conventional methods for extracting secondary metabolites and is widely used due to its ability to isolate soluble compounds from solid samples efficiently and reproducibly. This technique allows continuous extraction through solvent recirculation, ensuring prolonged contact with the sample and improving process yield without the need for large volumes of fresh solvent. In addition, it operates under controlled temperature conditions, preventing thermal degradation of sensitive compounds [27,28].

Additionally, a solvent system of increasing polarity (dichloromethane, ethanol, and water) was also used to determine the kind of secondary metabolites extracted according to their polarity. In this study, the highest yield was obtained in the following order of solvent: water, ethanol, and dichloromethane. However, in some methods, ethanol and dichloromethane had similar yields. Commonly, the bark and flowers of the Magnolia genus have been reported with a high presence of secondary metabolites [12,13,14]. In this study, fruit also has a high presence of secondary metabolites; therefore, this part of the plant could be proposed as a new source of molecules with pharmacological potential.

According to previous reports, the genus Magnolia contains more than eight hundred secondary metabolites primarily comprising alkaloids, terpenes, and phenols with diverse biological activities [29]. However, magnolol and honokiol are the main secondary metabolites associated with their anticancer activity. Therefore, in this study, both secondary metabolites were acquired as analytical standards to determine their absolute concentration in the extracts obtained. Honokiol had a retention time of 1.6 min, and magnolol had a retention time of 3.5 min. The retention time is a parameter that depends on the specific condition and chromatographic method used; for example, previous reports using HPLC methods show longer retention times, 7 and 10 min for honokiol and magnolol, respectively [30].

Analyzing the secondary metabolite profile of the extracts obtained, three hundred and twelve molecules were identified, as previously reported in the PubChem database. Magnone A, 2,3,5,8-tetramethyldecane, magnolialide, salicifoliol, pinoresinol, and yangambin were detected more frequently. Magnone A and pinoresinol are found in greater amounts in bark; 2,3,5,8-tetra-methyldecane, magnolialide, and salicifoliol are found more frequently in fruit; and yangambin is found in all three parts but in greater amounts in bark. Surprisingly, honokiol and magnolol were not detected in any extract. These results may be due to the LOD and LOQ obtained in the chromatographic method. Therefore, it will be necessary to analyze the extracts using a more sensitive analytical technique to confirm the absence or presence of both secondary metabolites in *M. alejandrae*. A study reports that, depending on the species of magnolia, different neolignan contents (% of total weight) can be obtained in ethanolic or methanolic extracts. For example, *M. officinalis* contains 1.00–1.25 of magnolol, 0.17–1.81 of honokiol, and 0.0003–1.24 of 4-O-methyl-honokiol, and obovatol was not detectable. In contrast, *M. obovata* contains 0.78–7.65 of magnolol, 0.55–1.25 of honokiol, 0.01–0.21 of 4-O-methyl-honokiol, and 0.01–0.33 of obovatol [31]. Interestingly, forty-eight secondary metabolites with an unreported molecular weight were detected, highlighting ten with a high frequency (Supplementary Material, Table S3 and Figures S3–S12). One secondary metabolite with the same molecular weight as honokiol and magnolol was detected, but with a shorter retention time (Supplementary Material, Figures S1 and S2). The results suggest that these secondary metabolites could be cryptic and/or novel. Therefore, in future studies, their isolation and characterization are necessary.

After, the nine extracts obtained with the highest yield using the Soxhlet conventional method were evaluated against five different cancer cell lines and one healthy cell line. *M. alejandrae* aqueous extracts of fruit and bark (FSW and BSW), although they did not contain detectable amounts of honokiol and magnolol, exhibited antiproliferative activity against the five cancer cell lines HeLa, MCF-7, A549, U373, and PC3. FSW and BSW had a high antiproliferative activity against the cancer lung cell line and BSW against the cancer prostate cell line (IC_50_ < 60 µg/mL). These results suggest a selectivity that could be related to the kind of secondary metabolites in each extract. Additionally, of the three leaf extracts obtained using the ultrasound non-conventional method, only organic extracts (LUE and LUD) had an antiproliferative activity against the five cancer cell lines; however, the concentrations were higher than those of FSW and BSW extracts, except for the LUE extract against the cancer cervical cell line. These results confirm that the part of the plant and the extraction method affect the obtention of secondary metabolites with antiproliferative activity. Finally, no extracts had antiproliferative activity against the non-cancerous MCF-10A cells.

The reference compounds magnolol and honokiol also caused antiproliferative activity against the five cancer cell lines with similar or equal values of IC_50_ to that of the anticancer doxorubicin drug. Interestingly, neither secondary metabolite had antiproliferative activity against MCF-10A at concentrations up to 150 µM; however, doxorubicin had an IC_50_ value of 1.5 µM. Honokiol and magnolol are secondary metabolites found in different species of the genus Magnolia, which have been shown to act effectively against cell proliferation in various types of cancer. This action is due to their ability to trigger programmed cell death processes through both the mitochondrial pathway and membrane receptors, which activate specific enzymes such as caspases and alter the balance between proteins that regulate cell survival. In addition, they interfere with the cell cycle by stopping its progress at key stages through the inhibition of regulatory proteins. These compounds also block cell growth and survival signals, interfering with pathways such as Akt, NF-κB, and MAPK. Another important aspect is that they promote the accumulation of reactive oxygen species, which creates an unfavorable cellular environment and can activate responses such as cycle arrest or apoptosis. Finally, magnolol and honokiol have been shown to reduce the formation of new blood vessels and limit metastatic potential by affecting factors such as VEGF and enzymes that facilitate cell migration [32,33,34,35,36]. Therefore, in future studies, it is necessary to identify the secondary metabolites of *M. alejandre* responsible for the antiproliferative activity to determine their mode of action.

The biological evaluation of extracts, magnolol, honokiol, and doxorubicin against cancer and normal cell lines allowed determining the selectivity index (SI). The aqueous extracts FSW and BSW show an SI value higher than 2 against the five cancer cell lines, highlighting activity against lung cancer. However, the organic extracts LUE and LUD showed a lower selectivity (SI < 2). Interestingly, magnolol and honokiol showed SI values higher than 15, except against prostate cancer cells. On the contrary, doxorubicin showed lower selectivity with SI values < 0.5. These results suggest a favorable and broad therapeutic window for the extracts, magnolol, and honokiol.

Despite promising results reported on various species of the Magnolia genus, the studies have significant limitations, such as the absence of clinical trials to validate their efficacy and safety in humans. These limitations highlight the need for more systematic and rigorous studies to support the therapeutic application of secondary metabolites from the Magnolia genus.

## 5. Conclusions

In this study, in organic and aqueous extracts from *M. alejandrae* were detected more than three hundred secondary metabolites previously reported in the Magnolia genus; their detection and concentration varied according to the solvent and extraction method used, highlighting the efficiency of the Soxhlet procedure. Additionally, these extracts contained forty-eight secondary metabolites, including cryptic compounds that need to be fully isolated and structurally elucidated. Four extracts exhibited antiproliferative activity that was not related to the detection of honokiol and magnolol. These results suggest their potential use of *M. alejandrae* as a source of bioactive compounds, although there are more necessary studies.

## Figures and Tables

**Table 1:** Chromatographic parameters of magnolol and honokiol standards detected by UPLC-MS.

Standard	Retention Time (Min)	Theoretical *m*/*z* [M+H^+^]^+^	Experimental *m*/*z* [M+H^+^]^+^	Majority Ion	LOQ	LOD
Honokiol	1.616	267.1413	267.62	267.62	0.025	0.0125
Magnolol	3.504	267.1379	267.61	267.61	0.025	0.0125

*m*/*z*: mass–charge, [M+H^+^]^+^: proton ion addition, LOD: limit of detection, LOQ: limit of quantification.

**Table 2:** Most frequent secondary metabolites detected in *M. alejandrae* extracts by UPLC-MS.

Secondary Metabolite	MW	Part of Plant	Method/Solvent											
			Maceration			Ultrasound			Soxhlet			Reflux		
			W	E	D	W	E	D	W	E	D	W	E	D
Magnone A	402.4	F	+	+	++	+	+	+	+	+	+	+	-	++
		L	+	+	+	+	+	-	+	+	+	+	-	+
		B	++	++	++	+	+	++	+	++	++	+	+	+
2,3,5,8-Tetramethyldecane	198.4	F	+	+	++	-	+	+	-	+	++	-	+	+
		L	-	-	+	+	++	-	+	+	++	+	-	-
		B	-	-	-	-	-	-	-	-	-	-	-	-
Magnolialide	249.1	F	+	+	++	+	++	++	++	+	++	++	++	++
		L	-	-	-	-	+	-	+	+	+	-	-	-
		B	-	+	++	-	++	-	-	++	+	-	+	++
Salicifoliol	250.2	F	+	+	+	+	++	++	++	+	++	++	++	++
		L	-	-	-	-	-	++	+	-	-	-	-	-
		B	+	+	++	-	+	+	-	+	+	+	+	-
Pinoresinol	358.4	F	+	+	++	+	+	+	-	-	+	+	-	++
		L	+	+	+	++	+	-	+	+	+	+	++	+
		B	++	++	+	+	+	+	-	++	++	++	++	++
Yangambin	446.5	F	+	+	++	+	+	+	+	+	-	+	-	++
		L	+	+	+	+	+	-	+	+	-	+	+	+
		B	++	++	+	+	++	+	-	++	+	+	++	+

MW: Molecular weight, W: Water, E: Ethanol, D: Dichloromethane, F: Fruit, L: Leaf, B: Bark, -: No detected, +: Low intensity (<10^6^ µV/s), ++: Medium intensity (10^6^–3 × 10^6^ µV/s).

**Table 3:** Most frequent unidentified secondary metabolites detected in *M. alejandrae* extracts by UPLC-MS.

SM	MW	Part of Plant	Method/Solvent											
			Maceration			Ultrasound			Soxhlet			Reflux		
			W	E	D	W	E	D	W	E	D	W	E	D
C	267.59	F	-	-	-	-	+	++	-	-	-	-	-	-
		L	-	++	-	-	+	-	-	-	++	+	-	-
		B	+	+	+	-	-	-	+	-	+	+	+	+
C1	401.73	F	+	+	+	-	-	-	-	+	-	-	+	+
		L	-	+	-	-	-	-	-	+	+	-	-	-
		B	+	+	-	-	-	+	+	-	+	+	+	+
C2	349.62	F	-	+	++	-	+	+	-	+	+	-	-	+
		L	-	-	++	-	+	-	-	+	+	-	-	+
		B	+	++	-	+	-	++	+	-	+	-	+	+
C3	440.80	F	-	-	-	-	-	-	+	+	+	+	+	-
		L	-	+	-	-	-	-	-	-	-	-	-	-
		B	-	-	-	-	+	-	+	-	-	-	-	-
C4	484.75	F	-	-	+	-	-	-	+	+	+	+	+	+
		L	+	-	-	-	-	-	-	-	+	-	-	-
		B	-	-	-	-	-	-	+	-	-	-	+	-
C5	453.54	F	++	-	-	-	-	-	+	+	-	-	-	-
		L	++	-	-	-	-	-	-	-	-	-	-	+
		B	+	-	-	-	-	-	+	-	-	+	-	-
C6	497.50	F	++	-	-	-	-	-	+	-	-	+	+	-
		L	++	-	-	-	-	-	-	-	-	-	-	-
		B	-	-	-	+	-	-	-	-	-	-	+	-
C7	541.44	F	++	-	-	-	-	-	-	-	-	+	+	-
		L	++	-	-	-	-	-	-	-	+	-	-	-
		B	+	-	-	+	-	+	+	-	-	-	-	-
C8	484.80	F	+	-	-	-	-	-	+	+	+	+	-	+
		L	+	-	-	+	-	-	-	-	-	-	-	-
		B	-	-	-	-	-	-	+	-	-	-	+	-
C9	481.55	F	-	-	+	-	-	-	-	+	-	-	-	++
		L	-	-	-	-	-	-	-	-	+	-	-	-
		B	-	+	-	-	+	-	-	-	-	-	++	-

MW: Molecular weight, W: Water, E: Ethanol, D: Dichloromethane, F: Fruit, L: Leaf, B: Bark. -: No detected, +: Low intensity (<10^6^ µV/s), ++: Medium intensity (10^6^–3 × 10^6^ µV/s).

**Table 4:** Half-maximal inhibitory concentration of *M. alejandrae* extracts (IC_50_ in μg/mL), magnolol, honokiol, and doxorubicin (IC_50_ in µM) against five cancer cell lines and one normal cell line.

Extracts	Cell Line IC_50_ (μg/mL)					
	HeLa (Cervical)	MCF-7 (Breast)	A549 (Lung)	U373 (Glioma)	PC3 (Prostate)	MCF10A (Normal Cell)
LSW	>300	>300	>300	>300	>300	>300
LSE	>300	>300	>300	>300	>300	>300
LSD	>300	>300	>300	>300	>300	>300
FSW	107 ± 27	130 ± 11	54 ± 13	101 ± 17	125 ± 17	>300
FSE	>300	>300	>300	>300	>300	>300
FSD	>300	>300	>300	>300	>300	>300
BSW	102 ± 49	137 ± 56	34 ± 14	67 ± 18	49 ± 5	>300
BSD	>300	>300	>300	>300	>300	>300
BSE	>300	>300	>300	>300	>300	>300
LUW	>300	>300	>300	>300	>300	>300
LUE	97 ± 42	235 ± 31	202 ± 32	249 ± 32	263 ± 34	>300
LUD	164 ± 78	161 ± 76	209 ± 76	283 ± 14	240 ± 48	>300
**Compounds**	**IC_50_ (µM)**					
Mag	4.9 ± 0.1	4.6 ± 3.6	5.5 ± 1.5	6.1 ± 3.4	17.8 ± 9.3	>150
Hon	4.7 ± 0.4	6.2 ± 2.6	9.6 ± 3.8	8.1 ± 2.8	21.5 ± 10.8	>150
Dx	4.6 ± 0.6	5.8 ± 0.5	4.8 ± 0.5	8.8 ± 5.5	15.6 ± 8.5	1.5 ± 1.2

IC_50_: half-maximal inhibitory concentration, LSW: Leaf–Soxhlet–Water, LSE: Leaf–Soxhlet–Ethanol, LSD: Leaf–Soxhlet–Dichloromethane, FSW: Fruit–Soxhlet–Water, FSE: Fruit–Soxhlet–Ethanol, FSD: Fruit–Soxhlet–Dichloromethane, BSW: Bark–Soxhlet–Water, BSE: Bark–Soxhlet–Ethanol, BSD: Bark–Soxhlet–Dichloromethane, LUW: Leaf–Ultrasonication–Water, LUE: Leaf–Ultrasonication–Ethanol, LUD: Leaf–Ultrasonication–Dichloromethane, Mag: magnolol, Hon: honokiol, Dx: doxorubicin. The data represent the mean ± S.D. of 3–4 independent determinations. Significant differences with the LSD test (*p* ≤ 0.05).

**Table 5:** Selectivity index of *M. alejandrae* extracts and the positive controls.

Cell Line	Extracts				Controls		
	LUE	LUD	BSW	FSW	Mag	Hon	Doxorubicin
HeLa	3.1	1.8	2.9	2.8	30.6	31.9	0.32
MCF-7	1.3	1.9	2.2	2.3	32.6	24.2	0.25
A549	1.5	1.4	8.8	5.6	27.3	15.6	0.31
U373	1.2	1.1	4.5	2.4	24.6	18.5	0.17
PC3	1.1	1.3	6.1	2.4	8.4	7	0.09

LUE: Leaf–Ultrasonication–Ethanol, LUD: Leaf–Ultrasonication–Dichloromethane, BSW: Bark–Soxhlet–Water, FSW: Fruit–Soxhlet–Water, Mag: magnolol, Hon: honokiol, Dx: doxorubicin.

## Data Availability

The original contributions presented in this study are included in the article/Supplementary Material. Further inquiries can be directed to the corresponding author(s).

## Supplementary Materials

The following supporting information can be downloaded at https://www.mdpi.com/article/10.3390/metabo15090567/s1, Graphic S1: Calibration curve of magnolol; Graphic S2: Calibration curve of honokiol; Figure S1: Chromatogram of new compound, honokiol (std), and magnolol (std) in the extract of leaves by Soxhlet with ethanol; Figure S2: Mass fragmentation spectrum of (a) new metabolite, (b) honokiol, and (c) magnolol by UPLC-MS; Figure S3: Mass fragmentation spectrum of C; Figure S4: Mass fragmentation spectrum of C1; Figure S5: Mass fragmentation spectrum of C2; Figure S6: Mass fragmentation spectrum of C3; Figure S7: Mass fragmentation spectrum of C4; Figure S8: Mass fragmentation spectrum of C5; Figure S9: Mass fragmentation spectrum of C6; Figure S10: Mass fragmentation spectrum of C7; Figure S11: Mass fragmentation spectrum of C8; Figure S12: Mass fragmentation spectrum of C9; Table S1: Secondary metabolites reported in *Magnolia* spp.; Table S2: The molecular weight of secondary metabolites unreported in the leaf, fruit, and bark of *Magnolia alejandrae*; Table S3: Identification of the unidentified secondary metabolites.
