# Supplementary material for: Phytochemical Analysis and Appraisal of Antiproliferative Activity of Magnolia alejandrae
Source: Metabolites. 2025 Aug 22;15(9):567. doi: 10.3390/metabo15090567 (PMC12472044; doi:10.3390/metabo15090567)
Supplement: Supplementary file 1 [file metabolites-15-00567-s001.zip › metabolites-3770272-supplementary.pdf]

Supplementary Material

Phytochemical Analysis and Appraisal of Antiproliferative Activity of *Magnolia alejandrae*

José E. Caballero-Chávez [1], Alma D. Paz-González[1], Diana V. Navarrete-Carriola [1], Fabián E. Olazarán-Santibañez[2], María Miriam Estevez-Carmona[3], Benjamín Nogueda-Torres[3], Fernando Emiliano Jiménez-Mondragón[4], Melany X. Márquez-Aguilar[4], Carmen Michelle Pineda-Alcala[4], Diego Cisneros-Juárez[4], Álvaro Marín-Hernández[4], Debasish Bandyopadhyay[5,6] and Gildardo Rivera[1,*]

[1] Laboratorio de Biotecnología Farmacéutica, Centro de Biotecnología Genómica, Instituto Politécnico Nacional, Reynosa, 88710, México; jcaballeroc2200@alumno.ipn.mx (J.E.C.-C.), apazg@ipn.mx (A.D.P.-G.), dnavarretec1900@alumno.ipn.mx (D.V.N.-C.)

[2] Facultad de Medicina Veterinaria y Zootecnia, Universidad Autónoma de Tamaulipas, Cd. Victoria, 87000, México; feolazaran@docentes.uat.edu.mx (F.E.O.-S.)

[3] Escuela Nacional de Ciencias Biológicas, Instituto Politécnico Nacional, 07320 Ciudad de México, México mmestevez@ipn.mx (M.M.E.-C.), bnogueda@ipn.mx (B.N.-T.)

[4] Departamento de Bioquímica, Instituto Nacional de Cardiología Ignacio Chávez, Ciudad de México,14080, México.; emi1920@comunidad.unam.mx (F.E.J.-M.), 317070589mema1920@iztacalacomunidad.unam.mx (M.X.M.-A.), carmenmichelle.pineda@ciencias.unam.mx (C.M.P.-A.), dieguito@ciencias.unam.mx (D.C.-J.), alvaro.marin@cardiologia.org.mx (A.M.-H.)

[5] School of Integrative Biological and Chemical Sciences (SIBCS), University of Texas Rio Grande Valley, Edinburg, TX 78539, USA debasish.bandyopadhyay@utrgv.edu (D.B)

[6] School of Earth, Environmental, and Marine Sciences (SEEMS), University of Texas Rio Grande Valley, Edinburg, TX 78539, USA

* Correspondence: giriveras@ipn.mx

Contents

1. Calibration curves of standards 2

2. Secondary metabolites reported in previous studies and databases in *Magnolia* ssp. 3

3. Detection of unidentified secondary metabolites 12

4. Detection of secondary metabolites with equal molecular weight that honokiol and magnolol 16

5. Identification data by UPLC-MS of the unidentified secondary metabolites 17

6. Spectral information of the unidentified secondary metabolites by UPLC-MS .. 18

1. Calibration curves of standards

1.1 Calibration Curve of Magnolol.

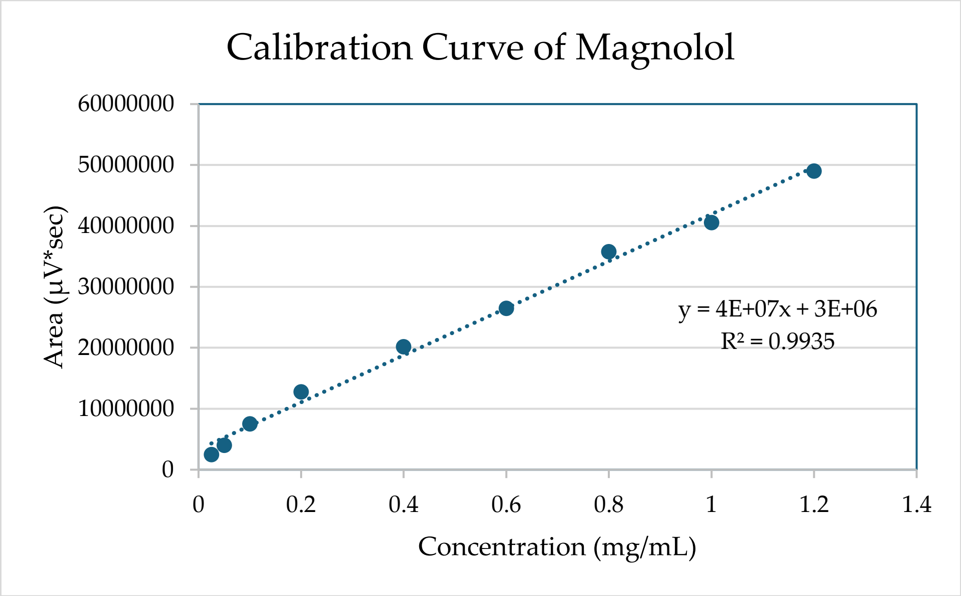

Graphic S1. Calibration Curve of Magnolol

1.2 Calibration Curve of Honokiol.

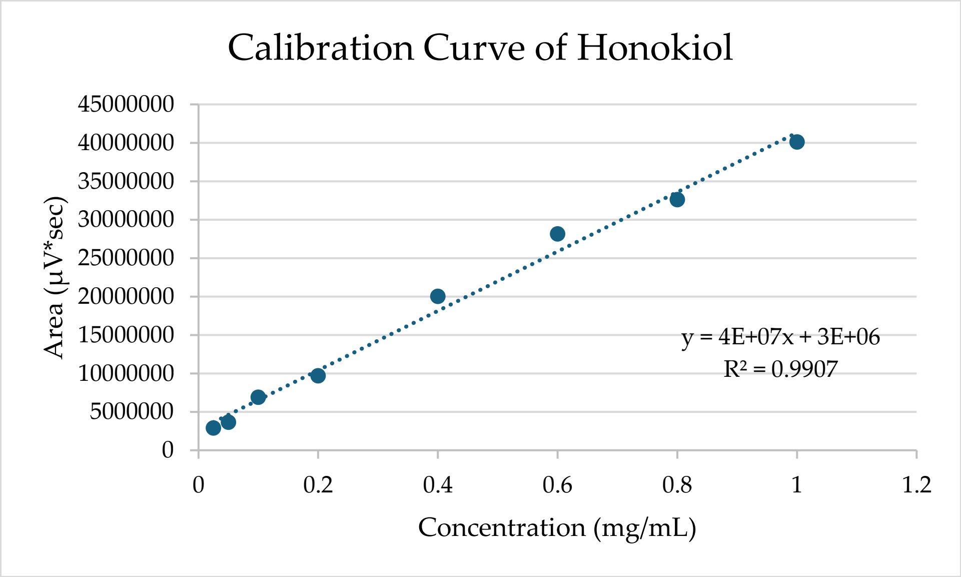

Graphic S2. Calibration Curve of Magnolol

2. Secondary metabolites reported in previous studies and databases in *Magnolia* ssp.

Table S1. Secondary metabolites reported in *Magnolia* spp.

No.	Compound	Classification	Molecular Weight (g/mol)
1	Anthranilic acid	Carboxylic acid	138.05
2	Phenylpyruvic acid	Carboxylic acid	165.05
3	Cinnamyl isovalerate	Carboxylic acid	219.13
4	2-Succinylbenzoate	Carboxylic acid	223.06
5	Cinnamyl cinnamate	Carboxylic acid	265.12
6	Coumaric acid	Hydroxycinnamic acid	164.16
7	Nature	Alkaloids	118.06
8	Tyramine	Alkaloids	137.18
9	3,6-Dihydroxynortropane	Alkaloids	144.1
10	3-Methyleneoxindole	Alkaloids	146.06
11	Crotanecine	Alkaloids	172.09
12	Saliciofolin	Alkaloids	210.29
13	Anonaine	Alkaloids	265.31
14	Mukoenine A	Alkaloids	266.15
15	Asimilobin	Alkaloids	267.32
16	(—)-Caaverine	Alkaloids	268.13
17	Liriodenine	Alkaloids	275.26
18	Roemerina	Alkaloids	279.3
19	1,3,11-Trihydroxy-2,9-dimethoxy-6,6-dimethylaporphinium	Alkaloids	280.37
20	Anolobin	Alkaloids	281.3
21	Lirinidine	Alkaloids	281.3
22	Nornuciferin	Alkaloids	281.3
23	Floribundine	Alkaloids	282.14
24	Xanthoplanin	Alkaloids	282.3
25	Amabiline	Alkaloids	284.18
26	Oxoanolobin	Alkaloids	291.26
27	Puterina	Alkaloids	296.12
28	Mecambrine	Alkaloids	296.12
29	Magnocline	Alkaloids	299.4
30	N-Methylcoclaurine	Alkaloids	299.4
31	N-Norarmepavina	Alkaloids	299.4

32	Lanuginosine	Alkaloids	305.3
33	N-Acetylanonaine	Alkaloids	308.13
34	Stefanina	Alkaloids	309.4
35	Nandigerina	Alkaloids	311.3
36	Armepavina	Alkaloids	313.4
37	(R)-Oblongin	Alkaloids	314.4
38	Lotusina	Alkaloids	314.4
39	Magnocurarine	Alkaloids	314.4
40	Corituberin	Alkaloids	327.4
41	O-Methylarmepvine	Alkaloids	327.4
42	Reticulin	Alkaloids	329.4
43	Dicentrinone	Alkaloids	335.3
44	Oxo-O-methylbulbocapnine	Alkaloids	340
45	Magnoflorina	Alkaloids	342.4
46	1,9,10-Trihydroxy-2-methoxy-6,6-dimethylaporphinium	Alkaloids	343.42
47	(S)-Tembetarine	Alkaloids	344.4
48	Isopiperolein B	Alkaloids	366.2
49	Magnolamide	Alkaloids	372.4
50	Magnofficina	Alkaloids	417.21
51	Dehydroemetine	Alkaloids	479.29
52	Magnolina	Alkaloids	596.7
53	Oxyacanthin	Alkaloids	608.7
54	Capillanol	Alcohol	175.11
55	3-[[5-Methyl-2-(1-methylethyl) cyclohexyl]oxy]-1,2- propanediol	Alcohol	253.17
56	Xanthorrhizol	Alcohol	271.06
57	Procyanidin B6	Alcohol	579.15
58	1-Phenylethylamine	Amines	122.09
59	Carboxynorspermidine	Amines	176.13
60	Phytosphingosine	Amines	318.29
61	Erythro-4-hydroxyphenylpropan-7,8-diol-7-o-ethylether	Aromatics	196
62	Dehydrodieugenol	Biphenyls	326.4
63	(6S)-dehydrovomifoliol	Ketones	223.13
64	Pyrogallol	Phenolic Compounds	127.03
65	Isoscopoletin	Phenolic Compounds	193.04
66	3beta-Dihydroxymarasmene	Phenolic Compounds	207.06
67	6-Hydroxy-5,7,4'- trimethoxyflavone	Phenolic Compounds	209.09
68	Isokaempferide	Phenolic Compounds	255.1

69	Chalcone	Phenolic Compounds	267.13
70	Lathodoratin	Phenolic Compounds	280.13
71	Isovalerate	Phenolic Compounds	284.18
72	Murrayacinine	Phenolic Compounds	301.06
73	Encecalin	Phenolic Compounds	310.18
74	Zanthosimuline	Phenolic Compounds	329.1
75	N-Methyl-N-(2-phenylethenyl)- 3-phenyl-2- oxiranecarboxamide	Phenolic Compounds	346.18
76	Magnoloside M	Coumarins	354.31
77	Scoparone	Coumarins	206.19
78	Coumanolignano	Coumarins	318.4
79	trans-Isomyristin	Phenylpropanoid	192.21
80	Sinapyl Alcohol	Phenylpropanoid	210.23
81	Deacylaserine	Phenylpropanoid	226.23
82	Sinapaldehyde	Phenols	208.21
83	Syringaresinol	Phenols	418.4
84	Cyanidin	Flavonoids	287.24
85	Isorhamnetin	Flavonoids	316.26
86	Vitexin	Flavonoids	432.4
87	Orientín	Flavonoids	448.4
88	buddlenoida A	Flavonoids	594.5
89	kaempferol 3-O-rutinoside	Flavonoids	594.5
90	Tiliroside	Flavonoids	594.5
91	Peonidin 3-rutinoside	Flavonoids	609.6
92	Peonidin 3,5-diglucoside	Flavonoids	661
93	Alhagidín	Flavonones	771.24
94	Piperitol	Furofurans	356.4
95	Matsutakic acid A	Glycosides	201.11
96	Salidroside	Glycosides	323.1
97	5'-O-beta-D-Glucosylpyridoxine	Glycosides	332.13
98	Magnolignan A-2-O-β-d-glucopyranoside	Glycosides	462.19
99	Isosyringinoside	Glycosides	534.5
100	Lividomycin B	Glycosides	600.31
101	Magnoloside F	Glycosides	609.184
102	Isoacteoside	Glycosides	623.19
103	Magnoloside C	Glycosides	623.19
104	Verbascoside	Glycosides	623.19
105	Magnoloside A	Glycosides	623.2
106	Magnoloside D	Glycosides	624.6

107	Polyumoside	Glycosides	770.7
108	Magnoloside B	Glycosides	785.25
109	(Z)-β-Ocimene	Monoterpene hydrocarbons	136.23
110	Camphene	Monoterpene hydrocarbons	136.23
111	Limonene	Monoterpene hydrocarbons	136.23
112	Sabineno	Monoterpene hydrocarbons	136.23
113	α-Phellandrene	Monoterpene hydrocarbons	136.23
114	α-Pinene	Monoterpene hydrocarbons	136.23
115	α-Terpinene	Monoterpene hydrocarbons	136.23
116	α-Terpinolene	Monoterpene hydrocarbons	136.23
117	α-Thujene	Monoterpene hydrocarbons	136.23
118	β-Myrcene	Monoterpene hydrocarbons	136.23
119	β-Pinene	Monoterpene hydrocarbons	136.23
120	γ-Terpinene	Monoterpene hydrocarbons	136.23
121	δ-3-Carene	Monoterpene hydrocarbons	136.23
122	Germacrene D	sesquiterpene hydrocarbons	204.35
123	α-Copaene	sesquiterpene hydrocarbons	204.35
124	α-Humulene	sesquiterpene hydrocarbons	204.35
125	α-Muurolene	sesquiterpene hydrocarbons	204.35
126	β-Caryophyllene	sesquiterpene hydrocarbons	204.35
127	δ-Cadinene	sesquiterpene hydrocarbons	204.35
128	β-Elemenone	sesquiterpene hydrocarbons	218.33
129	(-)-syringaresinol 4'-O-β-d-glucopyranoside	lignan glucoside	580.58
130	Randaiol	Lignans	242.27
131	Randainol	Lignans	281.12
132	Obovatal	Lignans	295.1
133	Guaiacín	Lignans	328.4
134	Sesamin	Lignans	330.3
135	Galbacín	Lignans	340.4
136	Episesamin	Lignans	354.4
137	Sesamin	Lignans	354.4
138	Calopiptín	Lignans	356.4
139	Machilusín	Lignans	356.4
140	Pinoresinol	Lignans	358.4

141	Fargesín	Lignans	370.4
142	Kobusin	Lignans	370.4
143	Sesaminon	Lignans	370.4
144	Filigenol	Lignans	372.4
145	Magnolenín C	Lignans	372.4
146	Sylvastemín	Lignans	372.4
147	Galgravín	Lignans	372.5
148	Veraguensín	Lignans	372.5
149	Epieudesmin	Lignans	386.4
150	Eudesmin	Lignans	386.4
151	Magnolone	Lignans	386.4
152	Magnostellin A	Lignans	388.5
153	Fargesol	Lignans	404.5
154	Epimagnolin	Lignans	416.5
155	Syringaresinol	Lignans	417.15
156	Magnostellin B	Lignans	418.4
157	Yangambin	Lignans	446.5
158	3-Cyclohexen-1-ol	Oxygenated monoterpenes	98.14
159	cyclohexylmethanol	Oxygenated monoterpenes	114.19
160	Camphor	Oxygenated monoterpenes	152.23
161	Eucalyptol	Oxygenated monoterpenes	154.25
162	Borneol acetate	Oxygenated monoterpenes	196.29
163	Magnatriol B	Neolignans	242.27
164	Magnaldehyde D	Neolignans	253.09
165	Magnaldehyde E	Neolignans	253.09
166	Magnaldehyde B	Neolignans	279.1
167	Magnaldehyde A	Neolignans	280
168	4-o-methyl honokiol	Neolignans	280.4
169	Saulangianín	Neolignans	280.4
170	Obovatol	Neolignans	282.3
171	Kachirachirol A	Neolignans	294
172	Magnolignano E	Neolignans	297.11
173	Magnolignano A	Neolignans	299.13
174	Magnolignano C	Neolignans	299.13
175	Liliflol A	Neolignans	310.3
176	Kachirachirol B	Neolignans	312
177	9-methoxyobovatol	Neolignans	312.36
178	Magnaldehyde C	Neolignans	314.3
179	Magnolignano B	Neolignans	315.12

180	Eupomatenoid-7	Neolignans	324.4
181	Licarín B	Neolignans	324.4
182	Licarín A	Neolignans	326.4
183	Liliflol B	Neolignans	326.4
184	Acuminatin	Neolignans	340.4
185	Burchellín	Neolignans	340.4
186	Denudatin A	Neolignans	340.4
187	Futoenone	Neolignans	340.4
188	Denudatin B	Neolignans	356.4
189	Dihydrodehydrodiconiferylalcohol	Neolignans	360.4
190	Fargesone C	Neolignans	370.4
191	Fargesona A	Neolignans	372.4
192	Fargesone B	Neolignans	372.4
193	Maglifloenone	Neolignans	386.4
194	Piperenone	Neolignans	388.5
195	Ashcantin	Neolignans	400.4
196	Magnosalin	Neolignans	416.15
197	Magnosalicin	Neolignans	432.5
198	Magnolignano I	Neolignans	522.6
199	Magnobovatol	Neolignans	534
200	Magnolignano H	Neolignans	562.6
201	Magnolignano F	Neolignans	564.7
202	Magnolignano G	Neolignans	596.7
203	Naphthalene	Others	128.17
204	Myristicin aldehyde	Others	180.16
205	Magnoshinin	Polyphenols	414.5
206	Magnolin	Polyphenols	416.5
207	Clovanemagnolol	Polyphenols	486.7
208	Eudeshonokiol A	Polyphenols	488.7
209	Eudeshonokiol B	Polyphenols	488.7
210	Eudesmagnolol	Polyphenols	488.7
211	Eudesobovatol A	Polyphenols	504.7
212	Eudesobovatol B	Polyphenols	504.7
213	Magnolianín	Polyphenols	827
214	Emodin	Quinone	127.05
215	γ-eudesmol	Sesquiterpenoids	222.4
216	Caryophyllene oxide	Sesquiterpenes	220.35
217	Costunolide	Sesquiterpenes	232.32
218	Parthenolide	Sesquiterpenes	248.32

219	Kachirachiraín	Sesquiterpenes	296.4
220	Elemol	Oxygenated sesquiterpenes	222.37
221	trans-Nerolidol	Oxygenated sesquiterpenes	222.37
222	α-Cadinol	Oxygenated sesquiterpenes	222.37
223	α-Eudesmol	Oxygenated sesquiterpenes	222.37
224	β-Eudesmol	Oxygenated sesquiterpenes	222.37
225	beta-Panasinsene	Terpenes	123.11
226	Massoia lactone	Terpenes	135.11
227	2-Propylthiophene	Terpenes	139.11
228	4-Acetyl-1-methylcyclohexene	Terpenes	153.12
229	Annuionone C	Terpenes	169.12
230	Heliannuol B	Terpenes	183.1
231	p-Cymene	Terpenes	191.1
232	Iridotrial	Terpenes	193.12
233	(R)-Hydnocarpic acid	Terpenes	193.15
234	Absinth	Terpenes	203.17
235	4-(2,6,6-Trimethylcyclohex-1-enyl)but-2-en-4-one	Terpenes	205.19
236	Rishitin	Terpenes	217.15
237	L-Menthyl acetoacetate	Terpenes	221.19
238	4,5-Dihydrovomifoliol	Terpenes	223.16
239	(+)-Perillyl alcohol	Terpenes	225.14
240	Germacrone-13-al	Terpenes	227.16
241	Carvacryl acetate	Terpenes	233.15
242	Atractylone	Terpenes	239.16
243	(3S,5R,6R,7E)-3,5,6-Trihydroxy-7-megastigmen- 9-one	Terpenes	241.17
244	Butylphthalide	Terpenes	249.14
245	Geranyl acetoacetate	Terpenes	265.14
246	Icacine	Terpenes	275.19
247	Spathulenol	Terpenes	294.18
248	alpha-Curcumene	Terpenes	428.2
249	Anacyclin	Terpenes	519.27
250	(1s,2r)-1-(4-Hydroxy-3-Methoxyphenyl)-2-(2-Methoxyphenoxy)propane-1,3-Diol	Lignans	320
251	Aureín	Alkaloids	335.4
252	Biondinín A	Alkaloids	374.4
253	Cariolanemagnolol	Lignans	486.7
254	β-Hydroxyacetoside	Phenolic compound	639.19

255	Hydron acetate	Fatty acids	60.05
256	Butanol	Alcohols	74.12
257	Butyric acid	Fatty acids	88.11
258	Valeric acid	Fatty acids	102.13
259	Magnolialide	Terpenes	249.1
260	Hexanoic acid	Fatty acids	116.16
261	Pentacyclo[4.2.0.02,5.03,8.04,7]oct-2-en-1-ol	Terpenes	118.13
262	Heptanoate	Fatty acids	129.18
263	2,6-Dimethyl-5-heptenal	Terpenes	140.22
264	4-Butylphenol	Phenolic compounds	150.22
265	d-Carvone	Terpenes	150.22
266	2,6-Dimethylnaphthalene	-	156.22
267	(S)-4-Nonanolide	Terpenes	156.22
268	Nonanoic acid	Fatty acids	158.24
269	1,5-Dihydroxynaphthalene	Phenolic compounds	160.17
270	Safrole	Phenolic compounds	162.18
271	Vanillic acid	Phenolic compounds	168.15
272	Dehydroconiferyl alcohol 9'-O-glucoside	Lignans	520.5
273	Methyleugenol	Phenolic compounds	178.23
274	syringaldehyde	Phenolic compounds	182.17
275	2,3,5,8-Tetramethyldecane	-	198.39
276	2-Propenal, 3-(4-hydroxy-3,5-dimethoxyphenyl)-	Phenolic compounds	208.21
277	Salicifoliol	Lignans	250.25
278	(8E)-4,9,12-trimethyl-3,14-dioxatricyclo[9.3.0.02,4]tetradec-8-en-13-one	Terpenes	250.33
279	Zingibertriol	Terpenes	256.38
280	Methoxymagnaldehyde E	Lignans	268.31
281	Higenamine	Alkaloids	271.31
282	4,4'-Dihydroxy-3,3'-dimethoxybenzophenone	Phenolic compounds	274.27
283	(12S)-3,5-dioxa-11-azapentacyclo[10.7.1.02,6.08,20.014,19]icosa-1(20),2(6),7,9,14,16,18-heptaen-13-one	Alkaloids	277.27
284	Diisobutyl phthalate	-	278.34
285	1,4-Dimethoxy-2-(4-methoxy-3-prop-2-enylphenyl)benzene	Phenolic compounds	284.3
286	Coclaurine	Alkaloids	285.34

287	Oxoanolobine	Alkaloids	291.26
288	Taxifolin	Flavonoids	304.25
289	Manglieside A	Lignans	312.31
290	5-(3-Methoxyprop-1-enyl)-3-(4-prop-2-enylphenoxy)benzene-1,2-diol	Lignans	312.4
291	O-Methylarmepavine	Alkaloids	327.4
292	Nectandrin B	Lignans	344.4
293	Oleiferin-f	Alkaloids	344.4
294	(2S,3S,4S)-2-(3,4-dihydroxyphenyl)-3,4-dihydro-2H-chromene-3,4,5,7-tetrol	Flavonoids	306.27
295	Pentacosane	-	352.7
296	1-(1,3-Benzodioxol-5-yl)-4-(3,4-dimethoxyphenyl)-2,3-dimethylbutan-1-ol	Lignans	358.4
297	Lariciresinol	Lignans	360.4
298	N,O-Diacetylmichelalbin	Alkaloids	365.4
299	Dichloroacetic acid	-	128.94
300	Taspine	Alkaloids	369.4
301	erythro-Guaiacylglycerol	Lignans	214.21
302	5-[(3S,3aS,6R,6aS)-6-(3,4,5-trimethoxyphenyl)-2,3,3a,4,6,6a-hexahydrofuro[3,4-b]furan-3-yl]-1,3-Benzodioxole	Lignans	400.4
303	Magnona A	Lignans	402.4
304	Stigmata-4,22-dien-3-one	Steroids	410.7
305	Stigmasterol	Steroids	412.7
306	Manglieside B	Lignans	444.4
307	beta-Amyrin acetate	Terpenes	468.8
308	Manglieside D	Lignans	522.5
309	Beta-Sitosterol 3-O-beta-D-galactopyranoside	Steroids	576.8
310	L-ascorbyl dipalmitate	Terpenoids	652.9
311	(2S,3R,4S,5S,6R)-2-[4-[(3S,3aS,6S,6aS)-6-[3,5-dimethoxy-4-[(2S,3R,4S,5S,6R)-3,4,5-trihydroxy-6-(hydroxymethyl)oxan-2 -yl]oxyphenyl]-1,3,3a,4,6,6a-hexahydrofuro[3,4-c]furan-3-yl]-2,6-dimethoxyphenoxy]-6-(hydroxymethyl)oxane-3,4,5-triol	Lignans	742.7
312	Magnolianin	Neolignans	827

3. Detection of unidentified secondary metabolites

Table S2. Molecular weight of secondary metabolites no reported in leaf, fruit and bark of *Magnolia alejandrae*.

Part of plant	Experimental m/z [M+H+]+
LEAF	70
	71
	75
	80
	86
	87
	92
	95
	95
	105
	105
	105
	117
	119
	152
	160
	213
	262
	266
	267
	307
	393
	394
	397
	438
	441
	454
	472
	477
	482
	485
	498
	529
	532
	541
	542

	569
	573
	585
	586
	613
	617
	628
	666
	680
	694
	971
	976
	1038
	1042
	1141
	1145
	1159
	1168
	70
	71
	74
	75
	80
	92
	95
	95
	105
	214
	215
	247
FRUIT	261
	262
	263
	288
	394
	397
	424
	438
	441
	454
	455
	456
	477

	482
	485
	490
	498
	526
	528
	529
	531
	534
	541
	542
	558
	569
	575
	585
	586
	587
	614
	620
	629
	630
	631
	664
	674
	688
	702
	703
	711
	718
	719
	726
	735
	746
	764
	897
	910
	920
	995
	1002
BARK	70
	74
	75
	80

87
95
95
102
105
160
163
215
258
259
262
394
397
438
441
454
477
482
485
497
498
509
526
531
532
533
541
542
569
573
585
586
620
631
702
729
755

4. Detection of secondary metabolites with equal molecular weight that honokiol and magnolol.

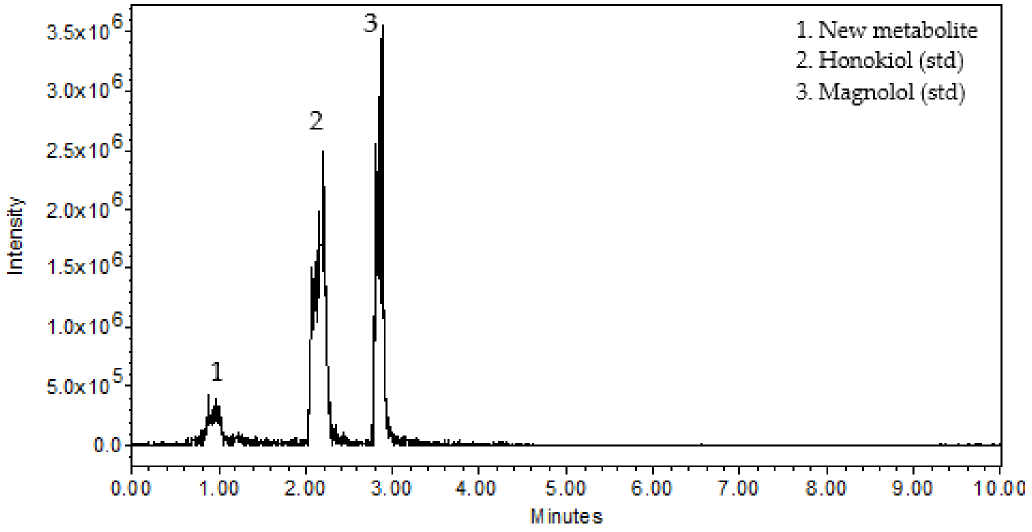

Figure S1. Chromatogram of new compound (1), honokiol (std), and magnolol (std) in the extract of leave by Soxhlet with ethanol.

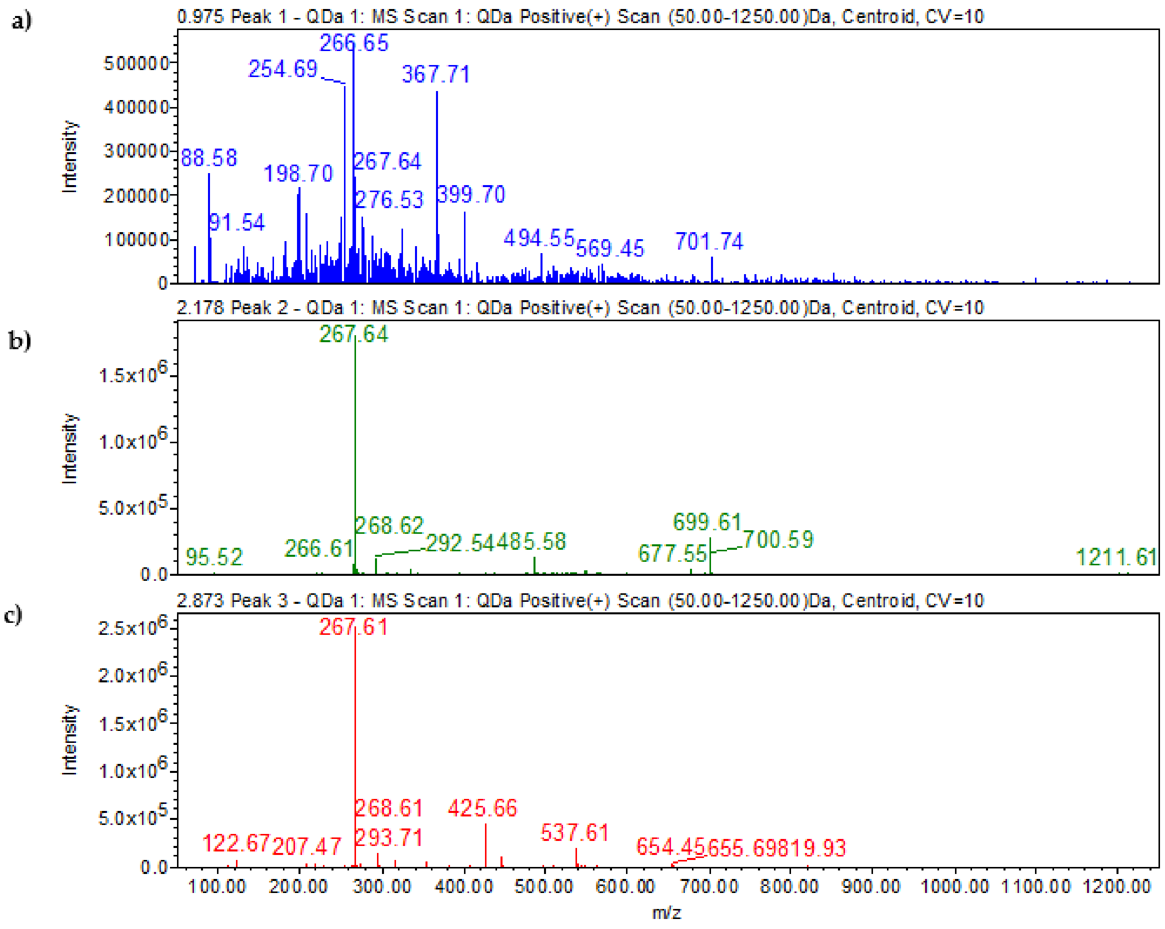

Figure S2. Mass fragmentation spectrum of a) new metabolite, b) honokiol and c) magnolol by UPLC-MS.

5. Identification data by UPLC-MS of the unidentified secondary metabolites.

Table S3. Identification of the unidentified secondary metabolites.

Compound	Retention time (min)	Experimental m/z $[M+H^+]^+$	Majority ion
C	2.574	267.59	267.59
C1	4.224	401.73	401.73
C2	1.164	349.62	102.58
C3	3.464	440.80	445.78

C4	3.442	484.75	489.65
C5	1.143	453.54	409.58
C6	1.147	497.50	409.57
C7	1.134	541.44	409.57/ 541.44
C8	4.199	484.80	484.80
C9	1.170	481.55	102.51

6. Spectral information of the unidentified secondary metabolites by UPLC-MS.

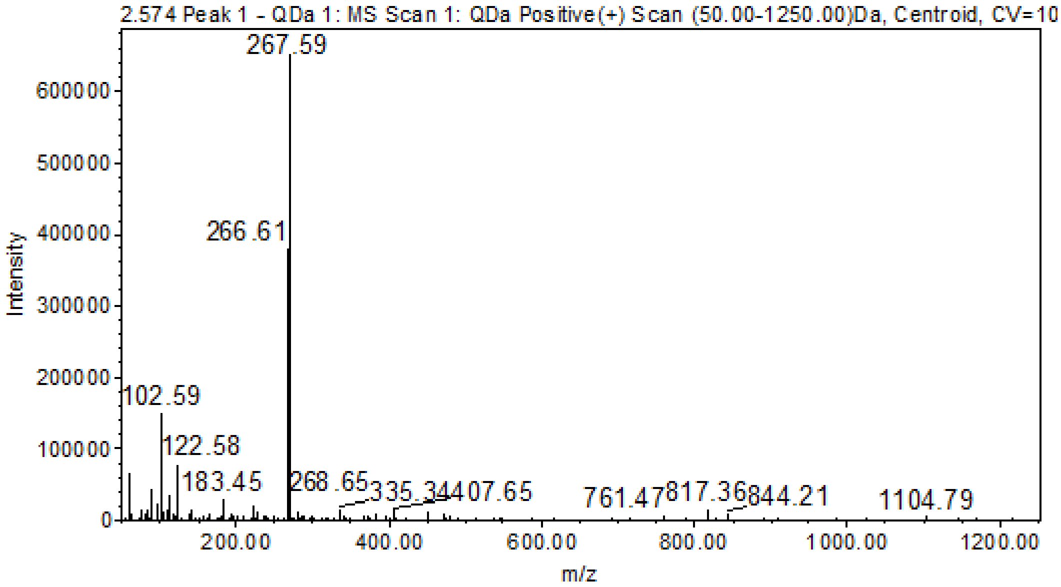

Figure S3. Mass fragmentation spectrum of C.

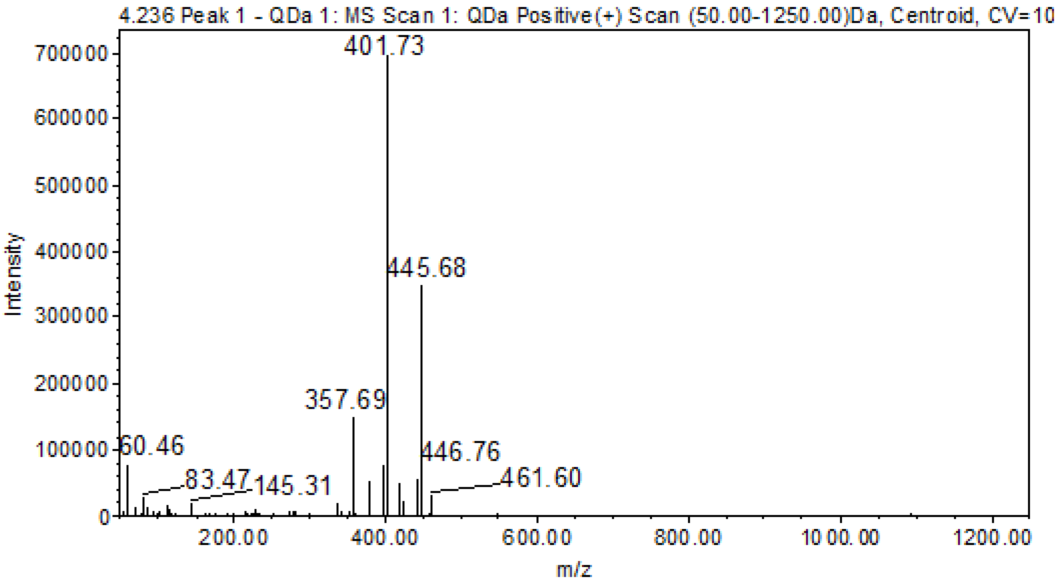

Figure S4. Mass fragmentation spectrum of C1.

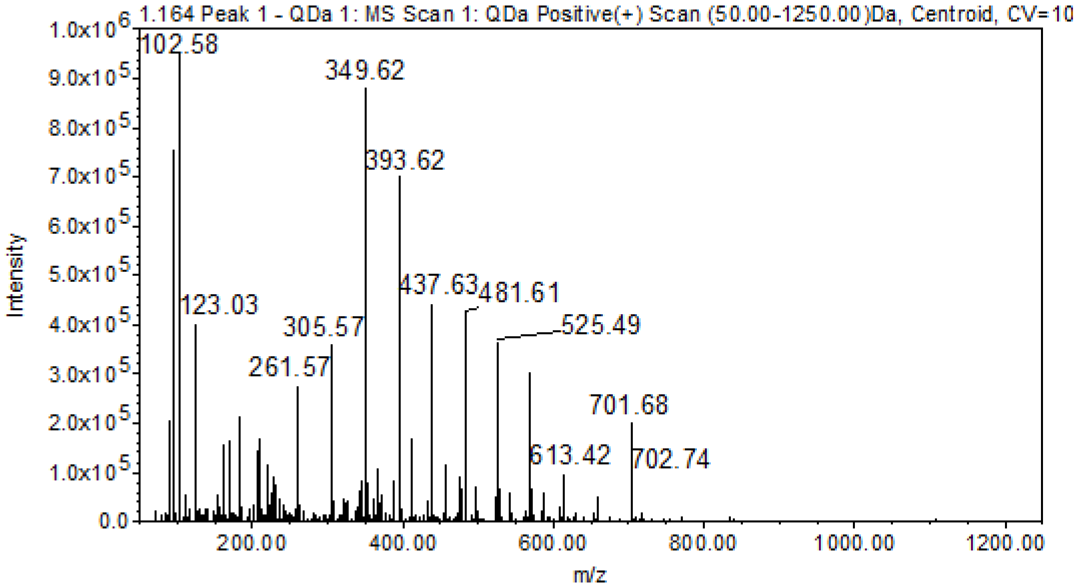

Figure S5. Mass fragmentation spectrum of C2.

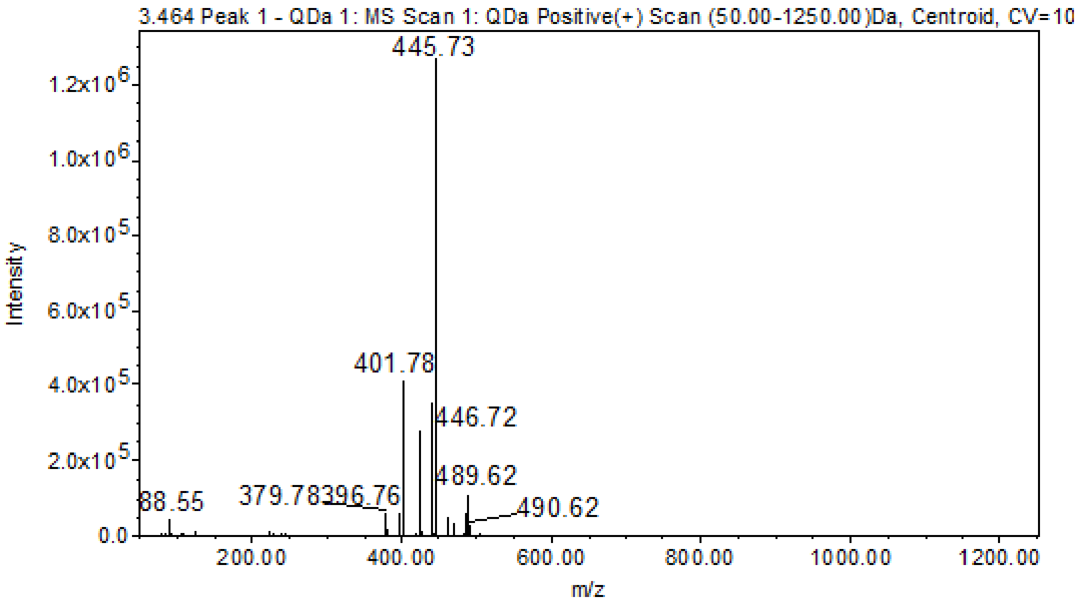

Figure S6. Mass fragmentation spectrum of C3.

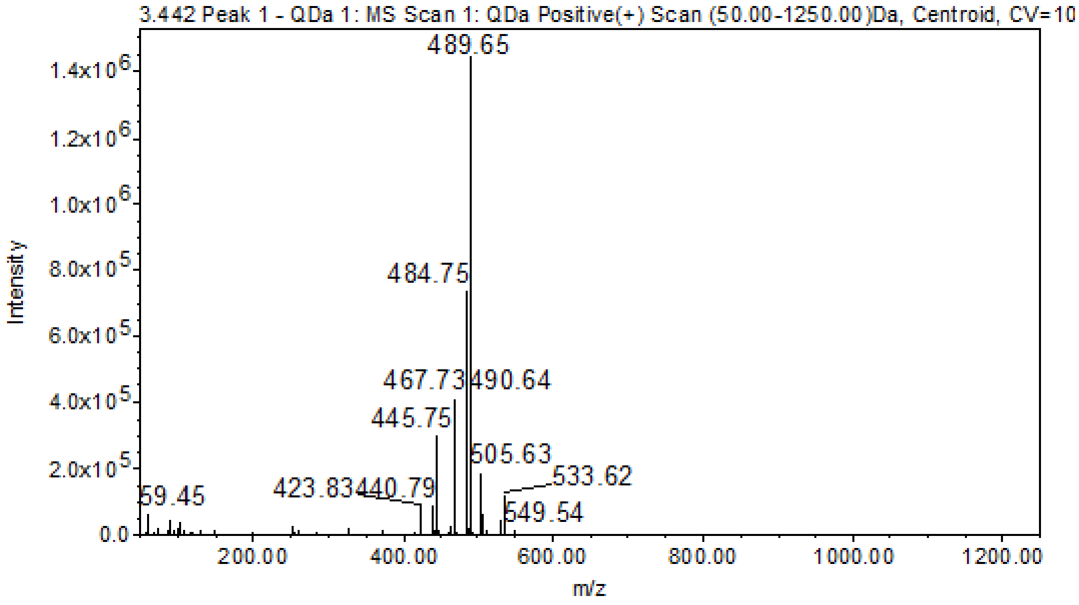

Figure S7. Mass fragmentation spectrum of C4.

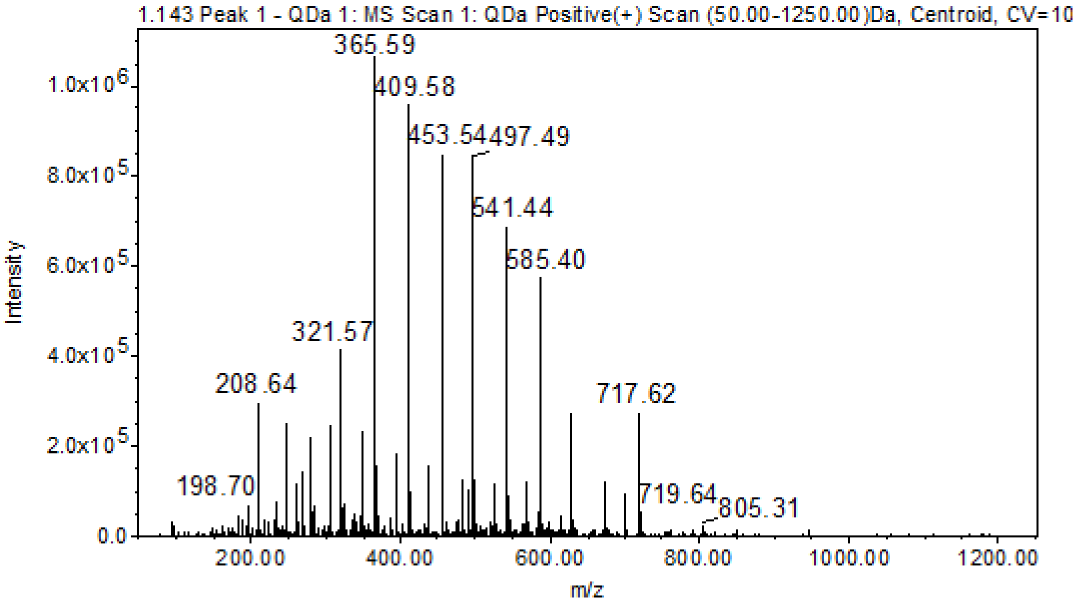

Figure S8. Mass fragmentation spectrum of C5.

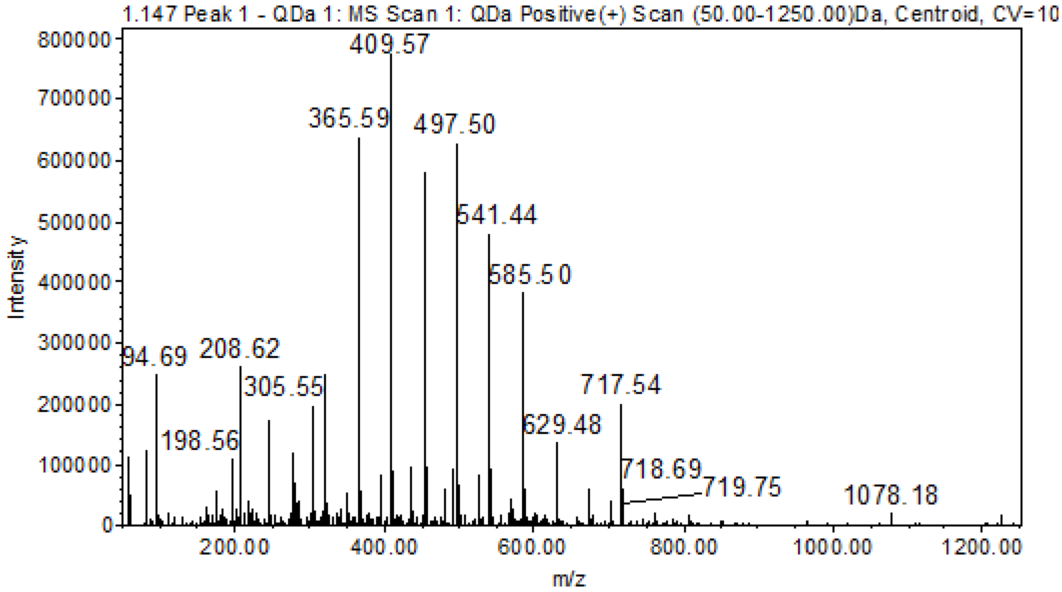

Figure S9. Mass fragmentation spectrum of C6.

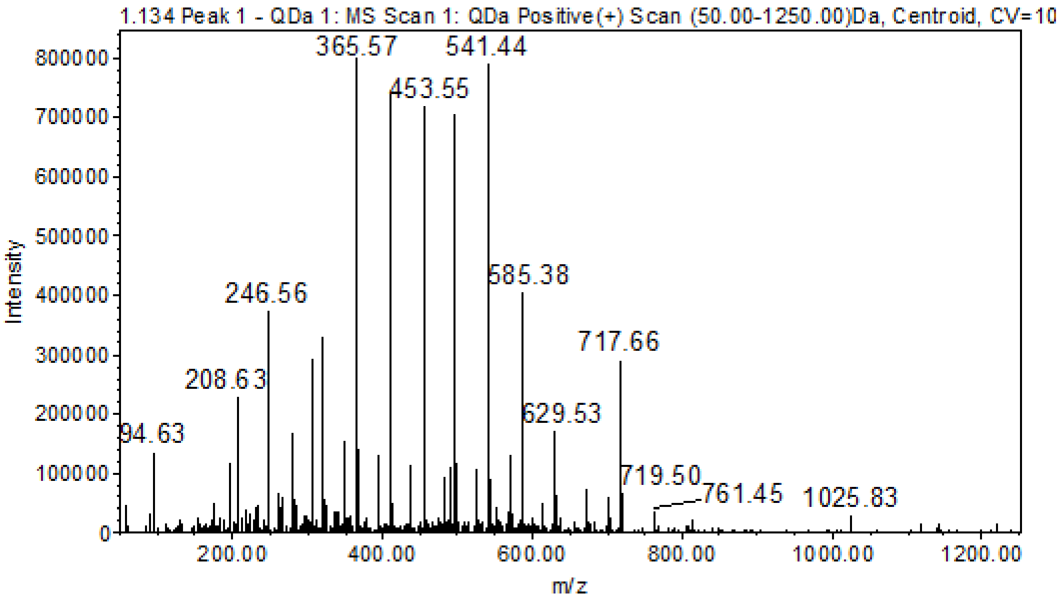

Figure S10. Mass fragmentation spectrum of C7.

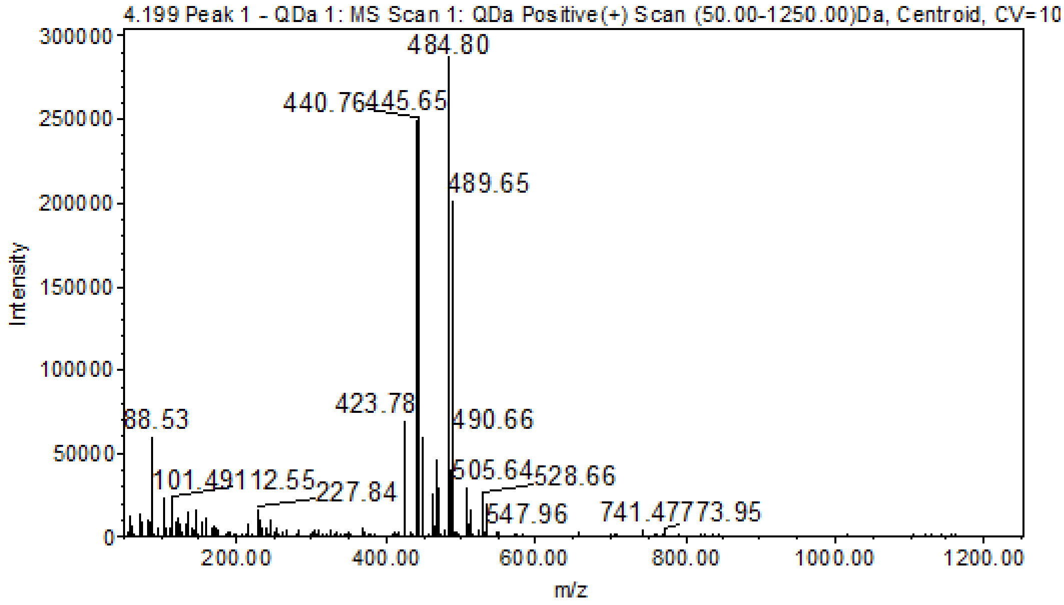

Figure S11. Mass fragmentation spectrum of C8.

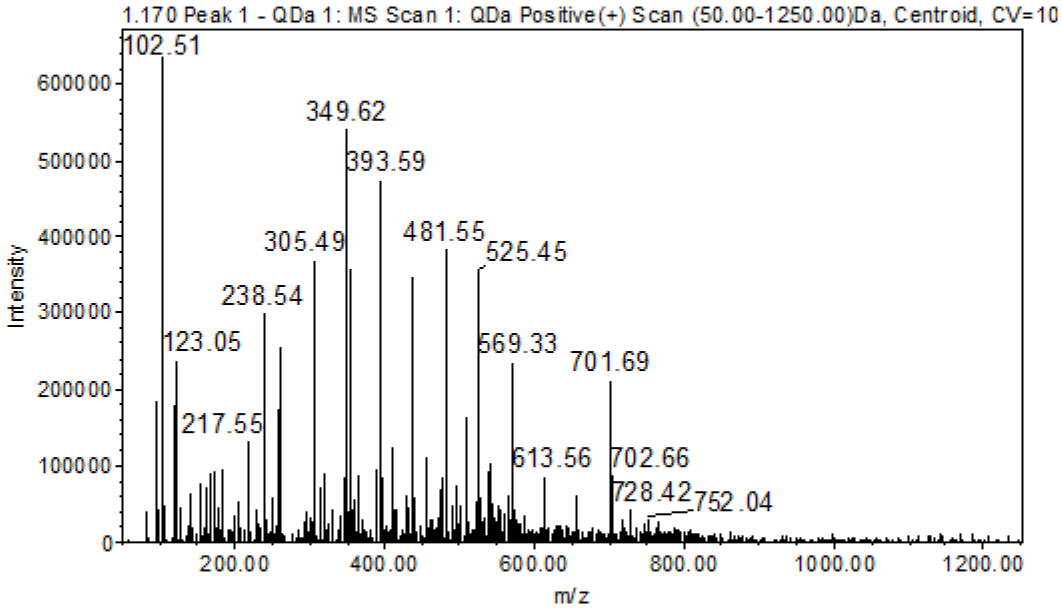

Figure S12. Mass fragmentation spectrum of C9.
